# Thermal analysis of flat plate solar air heater system with radiation reflectors and W-shaped roughness: artificial neural network & machine learning approach

**DOI:** 10.1038/s41598-026-41922-4

**Published:** 2026-03-02

**Authors:** Piyush Kumar Jain, Kawal Lal Kurrey, Vikas Pandey, Jitesh R. Shinde, Abhishek Narayan Tripathi, Niraj Kumar Dewangan

**Affiliations:** 1 Department of Mechanical Engineering, Bansal Institute of Science & Technology, Bhopal, India; 2 Department of Industrial and Production Engineering, School of Studies of Engineering & Technology, Bilaspur, India; 3 Department of Electrical Engineering, Babu Banarasi Das University, Lucknow, Uttar Pradesh 226028 India; 4 Department of Electronics Engineering (VLSI Design & Technology), CSMSS Chh. Shahu College of Engineering, Chh. Sambhajinagar (Aurangabad), Maharashtra India; 5 School of Electronics Engineering, Vellore Institute of Technology, Vellore, Tamil Nadu India; 6 Manipal Institute of Technology, Manipal Academy of Higher Education, Manipal, India

**Keywords:** Solar air heater, Radiation reflectors, W-shaped roughness, Artificial neural network, Machine learning, Thermal efficiency, Nusselt number, Energy science and technology, Engineering, Mathematics and computing, Physics

## Abstract

The lower thermal behavior of solar-based thermal systems limits the contribution of solar systems to meet current energy demand of industries. The Flat Plate Solar Air Heater (FPSAH) is extensively utilized in many applications requiring reasonable heat but struggles from inherent limitation in convective heat release and mediocre efficiency. In this study, the challenges are addressed with a novel means of dual mode augmented technique. This mode integrated absorber surface of the FPSAH system by introducing two radiation reflectors on either edge of the rectangular channel. Primarily these reflectors forward back the solar irradiance over the absorber plate, thus increasing the actual solar flux. Simultaneously, a W-shaped artificial rib roughness pattern is merged on the underneath (air-side) of the absorber plate. This coarseness is intended to persuade measured flow disorder inside the channel that disrupt the boundary layer development and may consequently augment convective heat transfer. Experimental testing is conducted with different combinations of roughened absorber surface and radiation reflectors. The performance enhancement is evaluated in terms of Nusselt number (*Nu*) and thermal efficiency of the FPSAH system. The maximum *Nu* achieved is 1.63 times higher using a set of radiation reflectors along with W-shaped roughness on the absorber surface compared to the plain configuration without radiation reflector. Finally, artificial neural network (ANN) and machine learning (ML) algorithms were used to predict Reynolds number in each set of experiments. A very good curve fitting was achieved by the Robust Regression algorithm with $R^2 = 0.99$ for the testing dataset and $R^2 = 0.94$ by the Random Forest Regression algorithm for ML.

## Introduction

The lower thermal features and wide use of solar-based thermal systems in moderate temperature applications draw attention for modifying the systems to give enhanced thermal performance^1−3^. The solar energy-based air heater, i.e., FPSAH system, is used for various low-temperature applications such as crop drying, space heating, and water distillation. The simple operational design, low maintenance cost, and silent operation with environmentally friendly behavior are the key advantages of the FPSAH system.

The main hindrance in using FPSAH is its low heat transfer (HT) coefficient between the absorber plate and flowing air. Conservation of solar radiation with maximum heat loss adversely affects thermal efficiency^4^. Utilizing solar air heaters contributes to lowering the overall carbon footprint of a building or process and aligns with global efforts toward sustainable energy solutions and climate action. A decreasing heat release rate elevates the plate’s temperature, thereby leading to increased heat losses to the atmosphere. Investigators have persistently attempted to enhance the HT coefficient of the FPSAH system through diverse methodologies, such as corrugated surfaces with V-patterns^5–7^, trapezoidal corrugated surfaces^8^, and extended surfaces (fins) to maximize thermal performance by increasing surface area for convective heat transfer^9–11^. However, these approaches often result in higher power consumption for air movement.

Several studies introduced artificial roughness in the form of ribs to generate turbulence adjacent to the heat transfer surface, minimizing friction while increasing turbulence inside the duct. Circular ribs were initially investigated^12^, followed by transverse patterns^13,14^, inclined patterns^15,16^, and V-patterns, which were found most efficient^17^. Modifications of V-patterns, such as discrete V-patterns, staggered elements, and gaps, have been extensively studied^11, 18–22^. Similarly, arc patterns and broken arc designs were explored^23,24^. Multi-pattern rib roughness configurations were also examined for thermal behavior improvements^25^.

Some approaches focused on modifying ducts using radiation reflectors to augment thermal characteristics. A square duct FPSAH system with radiation reflectors showed an increase in HT coefficient and Nusselt number up to 44% compared to a plain configuration^26^. The W-pattern of roughness demonstrated better thermal performance than simple V-patterns. W-shaped ribs provide flow separation, reattachment, and secondary flow generation. At lower flow velocities or Reynolds numbers ($Re > 4000$), flow conditions are insufficient to distort the thermal boundary layer, reducing heat discharge^27^. Selecting an optimum attack angle ($\alpha$) of $60^\circ$ in W-shaped ribs enhances secondary vortices and critical flow behavior near the surface, improving thermal performance^27^. W-pattern roughness also reduces pressure drop compared to transverse patterns.

A single-pass FPSAH system using rib roughness and reflectors was experimentally examined, showing increased thermal efficiency with higher air mass flow rates^28^. Studies on solar towers with Rankine cycles using thermodynamic laws and numerical techniques in GNU Octave have also been reported^29^. The collective effort to conclude various approaches of using rib roughness in FPSAH systems were conducted^22, 50^ to identify the scope to conduct the study in prescribed area of solar dryer systems and to observe the different approaches to proceed the studies further. From the literature, it is evident that no effort has been made to combine artificial roughness with radiation reflectors for FPSAH systems. Radiation reflectors redirect solar rays toward the absorber surface, raising its temperature, while artificial roughness interrupts thermal boundary layer formation, enhancing convective heat transfer. Numerous methods to expect the thermal behavior of solar dryer^35,40^ were also implemented earlier in many studies. In addition to above research work a different aspect of filling nano fluid under different climatic conditions were also conducted to analyze FPSAH system^51^.

The novelty of research lies in the cooperative integration of distinct thermal enhancement techniques specifically, W-shaped rib roughness and radiation reflectors to investigate their combined impact on convective-radiative heat transfer performance. This approach addresses the complex interplay between fluid-side convective augmentation and radiative heat transfer control, a combination that has not been extensively studied in a unified manner. Rib roughness, a passive heat transfer augmentation technique, is widely recognized for its ability to disrupt thermal boundary layers and induce turbulence, thereby increasing the Nusselt number (Nu). The Nusselt number is a dimensionless ratio quantifying the enhancement of heat transfer due to convection relative to pure conduction. Additionally, artificial neural networks (ANN) and machine learning (ML) algorithms are employed to predict Nusselt number variations, making the study more effective and innovative. In recent years, ANN and ML have gained prominence in engineering applications.

The key objectives of present research work are to broadly examine the shared influence of radiation reflectors over the absorber plate roughened with W-shaped rib roughness on the convective-radiative heat discharge augmentation in FPSAH system. It also demands a detailed analysis over W shaped rib rough absorber plate that how it contributes in enrichment of Nusselt Number (Nu). Another equally important objective is to develop and implement healthy extrapolative models for Nusselt number disparities using advanced machine learning (ML) algorithms, particularly artificial neural networks (ANN).

## Machine learning (ML)

Machine Learning (ML) has been widely applied in various engineering domains. For instance, ML is used in welding processes such as friction stir welding for classification and defect examination, and in resistance spot welding to predict mechanical properties^30–32^. Various heat transfer processes have been optimized using robust method optimization techniques^33,34^.

The multi-layer perceptron (MLP) architecture of neural models is among the most utilized in solar air heater applications^35^. For solar thermal collectors, several ML techniques such as Random Forest and Support Vector Regression have been employed for predicting hourly energy output^36^. ML algorithms empower machines to learn directly from data rather than relying on predefined equations^37^.

ML approaches are broadly categorized into two branches: supervised learning and unsupervised learning. Supervised learning includes classification and regression, while unsupervised learning covers clustering applications. In recent decades, ML algorithms have expanded significantly across various fields. Ensemble learning, which combines predictions from multiple ML algorithms, often yields more accurate results than a single model^38^.

Robust Regression is a type of regression analysis designed to overcome limitations of traditional parametric and non-parametric methods. It serves as an alternative to least squares, particularly when influential observations distort the data. This method attempts to determine the relationship between independent and dependent variables^39^.

In this study, both Random Forest and Robust Regression techniques are employed to predict Nusselt number (*Nu*) values during experiments.

## Artificial neural network (ANN) modelling

A neural network is a computational model that mimics the hierarchical structure of neurons by connecting nodes in layers^40^. It organizes information into conceptual levels, and its activity is defined by the connectivity and significance of these connections. During training, the weights are updated periodically according to a learning algorithm until the network performs the task accurately.

**Fig. 1:**
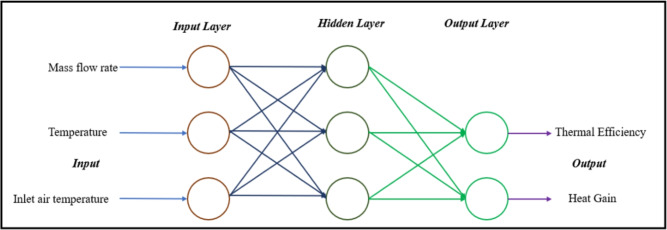
ANN architecture used for predicting *Nu* and $\eta _{th}$.

The ANN approach is highly effective for problems involving nonlinear and complex relationships among variables. In the Multi-Layer Perceptron (MLP) architecture, neurons are arranged into layers: an input layer, an output layer, and one or more hidden layers. These layers consist of interconnected neurons^41^, as illustrated in Fig. 1.

One of the objectives of this research is to develop an ANN model capable of predicting the Nusselt number (*Nu*) and thermal efficiency ($\eta _{th}$) in a heat exchanger as a function of various parameters.

## Details of experimental testing

### Experimental test facility

**Fig. 2:**
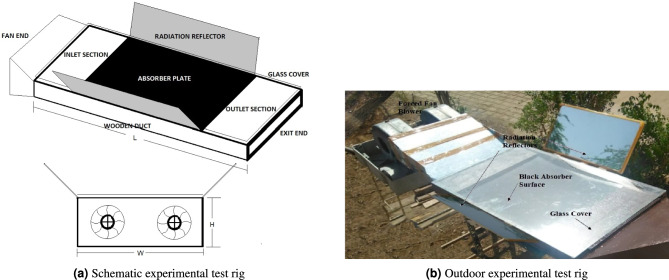
(**a**) Schematic experimental test rig (**b**) Outdoor experimental test rig.

The experimental analysis of W-shaped rib roughness with radiation reflectors was performed using an outdoor test rig shown in Fig. 2a. The measurement of the rectangular duct was obtained using ASHRAE standards^42^. Plywood of 18 mm thickness was used to fabricate the sidewalls of the SAH duct, which were insulated externally because of its cost effectiveness and ease of fabrication with a required property of insulation. The top of the duct was covered with an absorber surface made of galvanized iron with dimensions of 600 mm $\times$ 500 mm and a thickness of 1.2 mm. An absorber plate with a thickness of 1.2 mm has a significant influence on both thermal inertia and heat removal characteristics due to its limited conductive properties. The volumetric heat capacity $(\rho C_p)$ of a 1.2 mm thick absorber plate is relatively low, resulting in reduced thermal inertia and a rapid temperature response to variations in solar irradiance^1^. Transparent glass was used to cover the absorber surface to reduce reflective solar radiation.

The duct design was modified by attaching radiation reflectors at the edges of the testing duct. The placement of reflectors at the edge minimizes the heat loss from the sides of absorber plate and also redirected the radiations. The reflectors at the edge may enable more even temperature spreading over the plate and augmented convective heat release by modifying edge effects associated to increased heat loss. The mirrored reflector frame was hinged to the base duct and could be adjusted at any angle to reflect maximum possible radiation onto the exposed surface of the FPSAH system. The length of the mirrored reflectors was maintained equal to the test section (600 mm) with a height of 300 mm. The length of mirrored reflectors is maintained the same as the test section i.e. 600 mm with a height of 300 mm to direct maximum possible solar radiations on the active surface of absorber plate. This would maximize the potential for solar energy collection across the full length of the heat transfer zone. The tilt angle is maintained as $30^\circ$ from the horizontal to receive the solar radiation after getting deflected from the reflectors, an optimum position of reflector is essential for redirecting maximum possible radiations over the absorber plate. The key factors that influence the angle are position of sun throughout the day and geometry of collector and reflector. The tilt angle was maintained at $30^\circ$ from the horizontal to receive solar radiation after deflection from the reflectors. A glass mirror of 0.3 mm thickness with a polished surface was used.

The absorber surface of FPSAH system combined with additional reflectors increases the solar flux over the flat surface as compare to the direct radiation alone, that improve the overall thermal characteristics of the surface. The reflectors redirect the solar radiations and distributed across the absorber surface uniformly. The even distribution of heat flux, lesser thermal gradients and better overall stability of system may possibly accomplish through optimum geometry of reflector.

As recommended in prior studies, the duct was designed with an entrance length of $5\sqrt{WH}$ and an exit length of $2.5\sqrt{WH}$^43^. The selected inlet length of 550 mm facilitates the development of a fully developed airflow profile, while the 275 mm outlet section incorporates a mixing chamber positioned after the calculated distance from the test section. The mixing chamber contains uniformly spaced baffles that enhance turbulence and ensure a uniform temperature distribution at the duct outlet^44^. Axial flow fans were employed to maintain forced convection across the prescribed mass-flow-rate range and were integrated with a speed-regulation controller to adjust airflow as needed. Table 1 summarizes the dimensional specifications and components utilized in the experimental setup.

**Table 1:** Dimensions of experimental test setup.

Duct sections	Dimensions (mm)
Inlet section	550
Test section	600
Exit section	275
Duct height (*H*)	25
Duct width (*W*)	500
Total duct length (*L*)	1425

## Procedure and measurement

The experimental testing using the W-shaped roughened absorber surface of the FPSAH system was performed on clear sunny days between 10:00 a.m. and 1:30 p.m. A pyranometer was used to measure the heat flux received by the absorber surface during the investigation. The axial flow fan produced forced convection with different mass flow rates inside the duct. Wind velocity at the exit end was measured using an anemometer. Temperature readings were taken at several points along the duct using calibrated T-type thermocouple wires, and the readings were recorded by a digital data logger. A dimmer stat linked with the fan controlled the mass flow rate of air inside the duct. All data were noted under steady-state conditions, defined as the state where the data does not change for a definite period at a given location.

The instruments used in the testing, such as the pyranometer and anemometer, were of standard make and model. Thermocouples were calibrated using a simple method by comparing the temperature of the same substance measured with a thermometer and a thermocouple connected to a digital temperature data logger. Additionally, the data obtained for the smooth absorber surface using similar instruments fell within the acceptable range, ensuring the standardization of instruments used.

## Experimentation

The experimental test facility was located in an open area free from obstacles to receive direct solar radiation. The experimentation was conducted in April; hence, the best-suited timing to obtain intense solar radiation was between 10:00 a.m. and 1:30 p.m. The experiment was conducted at Rajasthan Technical University, Kota (Rajasthan), located at a latitude of $25^\circ 9^{\prime}$ North and longitude $75^\circ 50^{\prime}$ East, with an elevation of 271 meters above sea level.

Air temperature at the inlet and outlet, along with the average temperature of the absorber surface, was utilized to assess various performance parameters. The study began with scrutiny of components and instruments used. The glass cover was cleaned, and all connections were checked before switching on the axial flow fan. The mirrored radiation reflectors were adjusted by tilting them to redirect maximum solar radiation onto the horizontal flat absorber surface. A dimmer stat linked with the fan was attuned to maintain the predefined flow proportion in the duct.

The data collected from the experimental investigation included inlet air temperature ($T_i$), average outlet air temperature ($T_o$), mean absorber plate temperature ($T_p$), heat flux of solar radiation (*T*), and wind velocity (*V*).

Initially, inlet and outlet air temperatures were noted to calculate the mean bulk temperature of air ($T_f$), which is the simple arithmetic mean of the inlet and outlet temperatures:

1 $$\begin{aligned} T_f = \frac{T_i + T_o}{2} \end{aligned}$$

The average plate temperature ($T_p$) was sensed at six different points along the centerline of the absorber plate. The Reynolds number (*Re*) was calculated using the air velocity (*V*) at the exit and the hydraulic diameter of the duct ($D_h$):

2 $$\begin{aligned} Re = \frac{V D_h}{\nu } \end{aligned}$$

where the hydraulic diameter is defined as:

3 $$\begin{aligned} D_h = \frac{2WH}{W + H} \end{aligned}$$

The flow rate in terms of mass ($\dot{m}$) was calculated as:

4 $$\begin{aligned} \dot{m} = \rho V A \end{aligned}$$

where *A* is the rectangular duct cross-sectional area, $A = WH$.

Using Eqs. (5) and (6), the air’s further usable heat gain ($Q_u$) and its heat transfer coefficient (*h*) were evaluated [45]:

5 $$\begin{aligned} Q_u = \dot{m} c_p (T_o - T_i) \end{aligned}$$

6 $$\begin{aligned} h = \frac{Q_u}{A_s (T_p - T_f)} \end{aligned}$$

Finally, the thermal performance parameter of the FPSAH system, the Nusselt number (*Nu*), was calculated as:

7 $$\begin{aligned} Nu = \frac{h D_h}{k} \end{aligned}$$

## Investigated roughness and flow parameters

The experimental investigation was performed with different configurations: plain absorber surfaces, roughened absorber surfaces, and radiation reflectors. The present work involves an experimental analysis of an FPSAH system roughened with a W-shaped artificial roughness design combined with radiation reflectors to augment the thermal characteristics of the system.

A galvanized iron (GI) sheet of 1 mm thickness was used to fabricate the roughness geometry and served as the absorber surface in the present experimental testing. Galvanized iron is steel coated with zinc oxide, which improves its properties and functionality such as durability and strength. The density ($\rho$) of steel ranges from 7.85 to 8.15 g/cm^3^. The thermal conductivity (*k*) of GI is 204.2 W/mK and specific heat (*C*) is 896 J/kg·K. Hence, the thermal diffusivity ($\alpha$) can be calculated as:

8 $$\begin{aligned} \alpha = \frac{k}{\rho C} = 84.18 \times 10^{-6}~\text {m}^2/\text {s} \end{aligned}$$

A circular copper wire having a cross-sectional diameter of 2 mm was used to design the roughness due to its higher thermal conductivity. The roughness was fabricated with basic geometrical parameters optimized in^21^. The basic geometrical parameters such as roughness pitch (*q*) of 20 mm and attack angle ($\alpha$) of $60^\circ$ were considered to design the roughness.

The dimensionless parameters considered in the present experimental testing are *q*/*h*, $h/D_h$, and $\alpha /90$. Similar values of geometrical parameters were used to fabricate the W-shaped roughness design^21^. The optimum parameters that give maximum rise in thermal features of the FPSAH system are $q/h = 10$, $h/D_h = 0.047$, and $\alpha /90 = 0.667$.

The copper ribs were first cut into pieces of prescribed length. The complete detailed design with optimum geometrical dimensions was marked on the surface of the absorber plate, and then copper pieces were glued over the surface to fabricate the roughened absorber plate for testing purposes.

**Table 2:** Details of investigated roughness and flow parameters.

Parameters	Value
Relative roughness height ($h/D_h$)	0.047
Relative roughness pitch (*q*/*e*)	10
Relative attack angle ($\alpha /90$)	0.667
Flow velocity of air (*V*)	1.94, 2.59, 3.24, 3.88 m/s
Density of air ($\rho$)	1.174 kg/m^3^
Reynolds number (*Re*)	6036, 8045, 10060, 12072

**Fig. 3:**
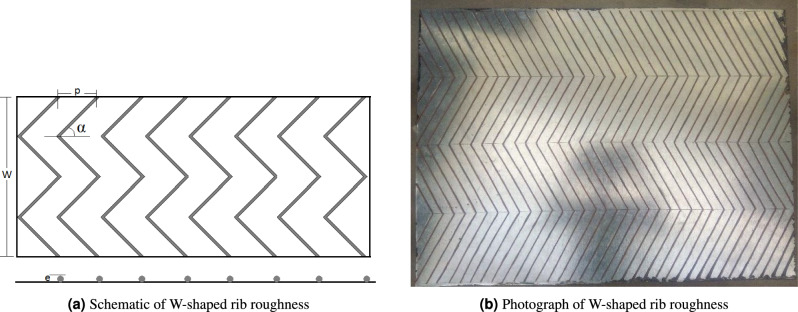
W-shaped rib roughness: (**a**) Schematic representation; (**b**) Photographic view.

The details of investigated parameters used in the design are presented in Table 2. The representation and detailed view of the W-shaped geometry are presented in Fig. 3a and b, respectively.

## Result and discussion

### Validation of experimental test rig

The experimental test rig, including a basic configuration of the absorber surface devoid of a radiation reflector, was subjected to ambient conditions. The thermal performance was assessed based on the Nusselt number (*Nu*) and the convective heat transfer coefficient ($h_{\text {exp}}$). The dimensionless parameter *Re* represented flow conditions inside the duct.

The theoretical value of *Nu* given by the Dittus-Boelter equation was compared with the *Nu* obtained from the experiment to validate the experimental data. The Dittus-Boelter formula is:

9 $$\begin{aligned} Nu = 0.023 Re^{0.8} Pr^{0.4} \end{aligned}$$

**Fig. 4:**
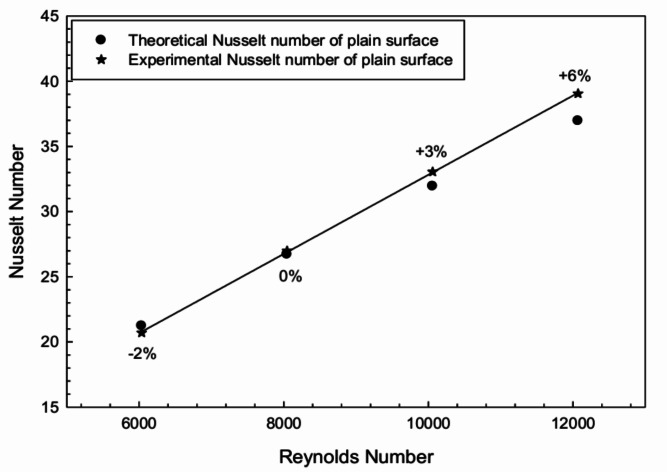
Comparative plot of theoretical and experimental Nusselt number.

The experimental values of *Nu* were compared with those obtained theoretically using Eq. (8) for the plain configuration of the absorber surface without radiation reflector. The comparison plot is shown in Fig. 4, and the values are found to be within a reasonable range of 6%, validating the experimental results.

Likewise, the frictional penalty or pressure drop during experimentation with the plain surface was calculated using the Modified Blasius equation:

10 $$\begin{aligned} f_s = 0.085 Re^{-0.25} \end{aligned}$$

**Fig. 5:**
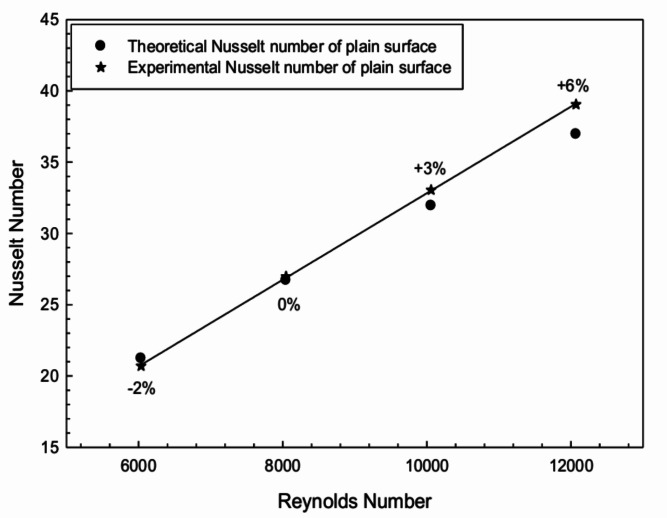
Comparative plot of theoretical and experimental friction factor.

The experimental values of *f* were compared with the theoretical values using Eq. (9) for the plain configuration. The comparison plot is shown in Fig. 5, and the values are found to be within a reasonable range of 10%.

The wind velocity (*V*) was obtained using an anemometer, and the corresponding air density ($\rho$) was used to measure the air flow rate ($\dot{m}$). The heat gain by the flowing air ($Q_u$) is calculated using Eq. (10)^21^, and the heat transfer coefficient ($h_{\text {exp}}$) is calculated using Eq. (11)^45^:

11 $$\begin{aligned} Q_u = \dot{m} c_p (T_o - T_i) \end{aligned}$$

12 $$\begin{aligned} h_{\text {exp}} = \frac{Q_u}{A_s (T_p - T_f)} \end{aligned}$$

where $T_f$ is the mean bulk air temperature, calculated as $(T_i + T_o)/2$, and $T_p$ is the absorber plate temperature.

The non-dimensional analysis of inlet air temperature and plate temperature was conducted using the absorber plate temperature coefficient ($C_{ta}$) defined as:

13 $$\begin{aligned} C_{ta} = \frac{T_p - T_f}{T_f} \end{aligned}$$

**Fig. 6:**
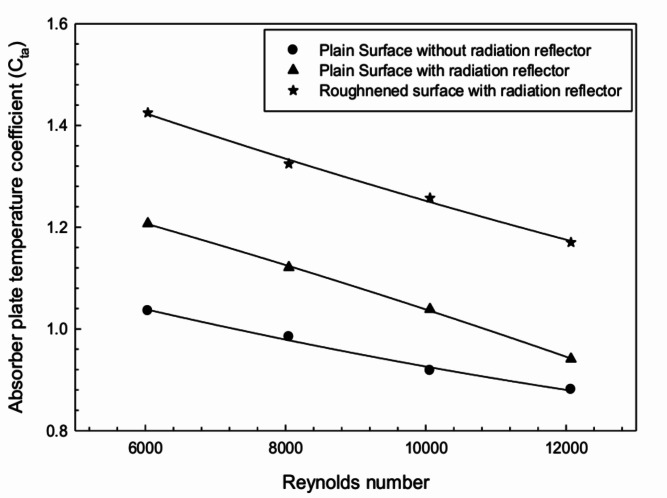
Variation of absorber plate temperature coefficient against Reynolds number.

The temperature coefficient ($C_{ta}$) of the absorber plate is plotted against *Re* in Fig. 6. The $C_{ta}$ value indicates the heat releasing characteristics of the hot absorber plate corresponding to air flow rate. It is observed that the average plate temperature reduces with a rise in *Re*, likely due to higher temperatures at lower flow rates. Increased flow rate results in an improved rate of heat release from the absorber surface.

It is also observed that the absorber plate gains higher temperature with the use of a radiation reflector compared to the plain configuration. A further increase in $C_{ta}$ is noted with the artificially roughened surface of the FPSAH system along with reflectors.

The heat received by the air across a small span around the absorber plate is used to calculate the thermal efficiency of the FPSAH system. The thermal efficiency is defined as the ratio of useful heat gained to total solar radiation incident on the absorber surface:

14 $$\begin{aligned} \eta = \frac{Q_u}{I A_s} \end{aligned}$$

### Uncertainty in results

The uncertainties in experimental results are estimated using the method proposed by Kline and McClintock (1953). The uncertainty in measurement is given by:

15 $$\begin{aligned} \delta y = \sqrt{\left( \frac{\partial y}{\partial x_1} \delta x_1 \right) ^2 + \left( \frac{\partial y}{\partial x_2} \delta x_2 \right) ^2 + \cdots } \end{aligned}$$

where $\delta x_1, \delta x_2, \ldots$ are uncertainties in individual measurements, and $\delta y$ is the total uncertainty.

The major uncertainties in the current study are:

- Nusselt number: 7.9% – 10.22%
- Reynolds number: 2.38% – 4.76%

### Influence of radiation reflectors

**Fig. 7:**
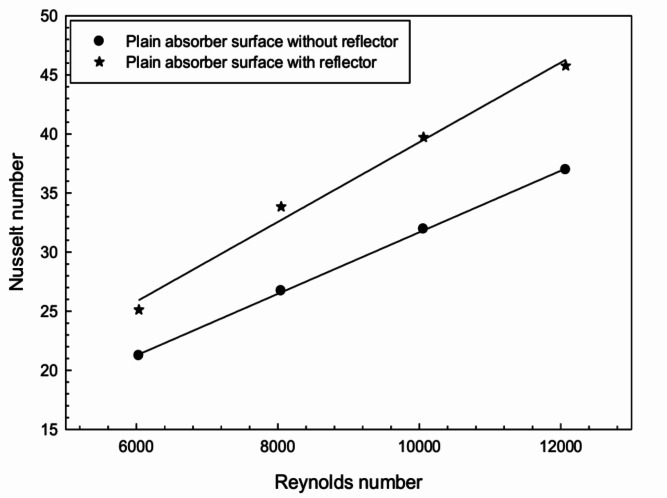
Effect of radiation reflector on Nusselt number with plain surface.

The influence of radiation reflectors on the thermal performance of the FPSAH system was analyzed. Reflectors were attached to the edges of the FPSAH system with a plain configuration. The outcomes in terms of *Nu* with and without radiation reflectors are plotted in Fig. 7. A considerable rise in heat transfer was observed with the use of radiation reflectors, with a percent increase in *Nu* ranging from 18 to 27%.

Radiation reflectors redirect more solar radiation toward the absorber surface, increasing its temperature and enhancing convective heat transfer to the air. The upper layer of flowing air gets heated by the hot absorber surface, and molecular exchange due to density differences improves thermal characteristics.

**Fig. 8:**
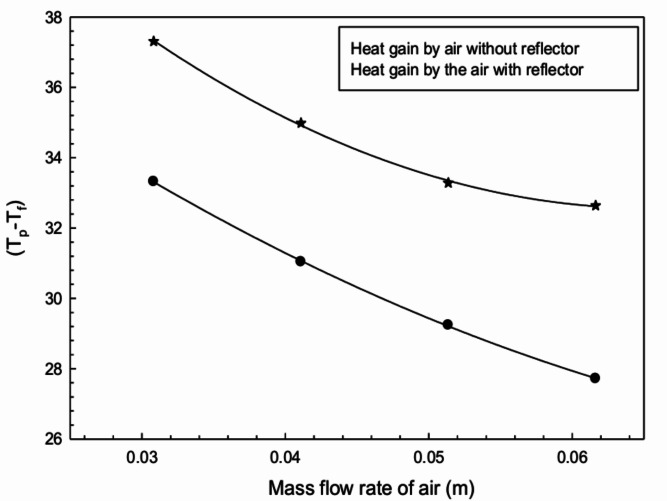
Heat gained by air with respect to mass flow rate.

To identify the effect of reflectors, the temperature difference $(T_p - T_f)$ is plotted against predefined air flow rates in Fig. 8. It is observed that air acquires significantly more heat using radiation reflectors under similar flow conditions.

**Fig. 9:**
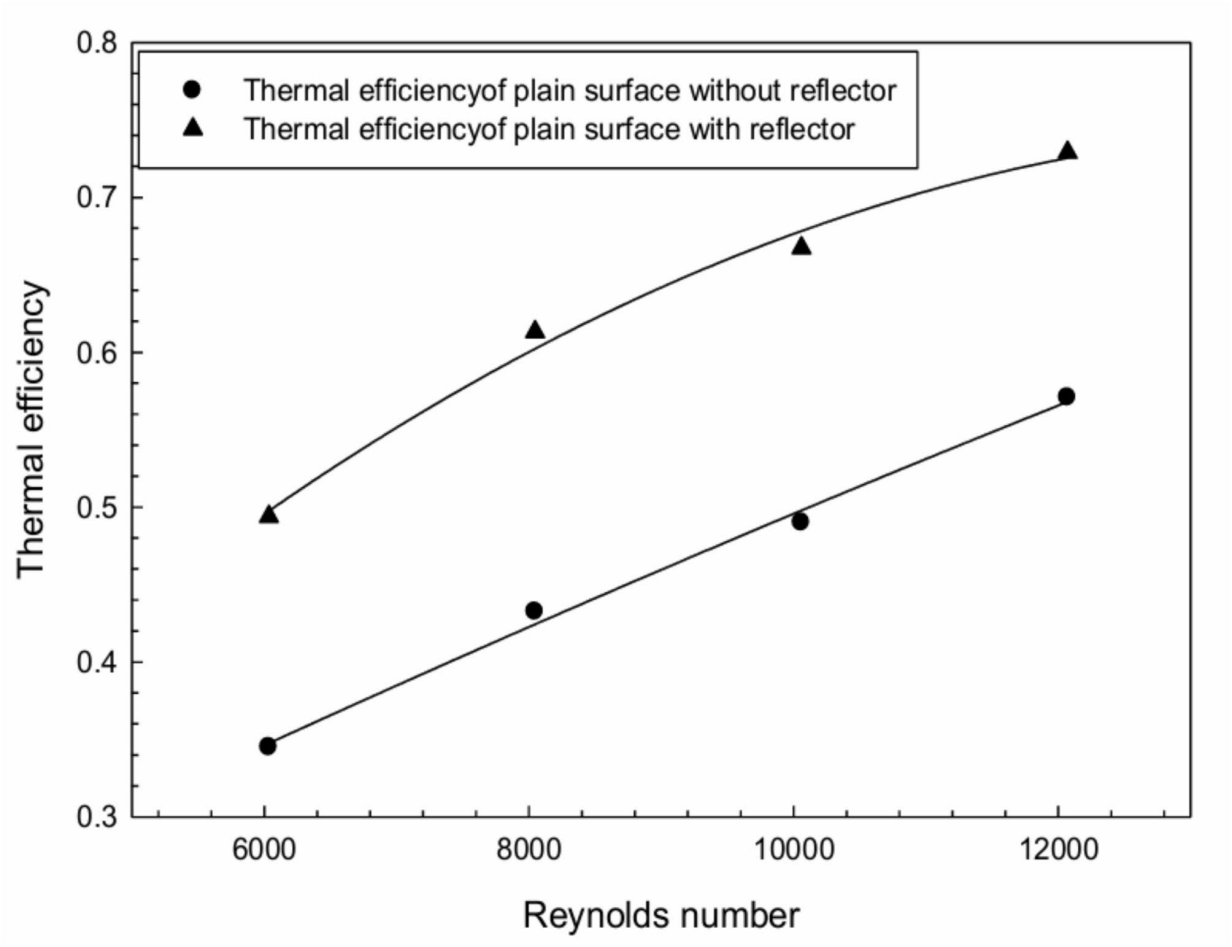
Thermal efficiency of plain configured surface with and without radiation reflectors.

Figure 9 shows the rise in thermal efficiency of the FPSAH system. The system with radiation reflectors and a plain absorber surface gains more heat compared to the system without reflectors. The thermal efficiency ranges from 1.27 to 1.43 times higher with reflectors.

### Influence of artificial roughness with radiation reflectors

The importance of using artificial roughness underneath the absorber surface has been explained in many earlier studies. The dimensions and geometrical arrangement of roughness were taken as recommended^21^. The relative roughness pitch (*q*/*h*) of W-shaped roughness was set at 10, with a rib inclination of $60^\circ$ from the edge of the plate.

W-shaped of rib roughness modifies the fluid flow characteristics. The multiple folds of rib roughness in W shape causes flow parting, reattachment of fluid flow and generation of secondary flow that improves the heat discharge form the surface. Apart from achieving better fluid flow behavior it is easy to design the shape of W from rib pieces with optimum heat release and minimum pressure drop.

The rib roughness elements interrupt the smooth flow of air and produce secondary flow and vortices inside the passage. The flow resistance leads to enhance the frictional behavior and hence pumping power. That can be reduced by considering optimum geometrical parameters such as relative roughness altitude $(e/D_h)$ where $D_h$ is hydraulic diameter of passage of fluid flow and relative roughness pitch (*P*/*e*). If heat transfer enrichment is significantly advanced than frictional penalty, implementing roughness is valuable. Proper optimization of roughness may augment heat discharge with minimum pumping loss.

**Fig. 10:**
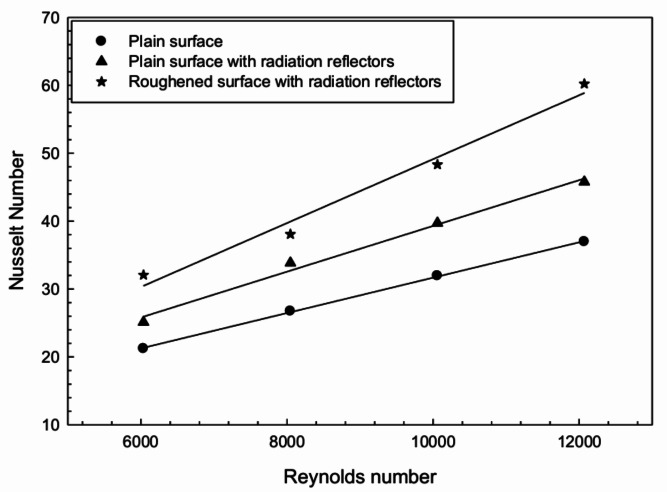
Nusselt number of different sets used in FPSAH system.

The implementation of artificial roughness design on the absorber surface along with radiation reflectors led to a significant improvement in the thermal performance of the FPSAH system. The maximum Nusselt number (*Nu*) for FPSAH with W-shaped roughness and radiation reflector was obtained as 1.63 times that of the plain configured FPSAH system without reflector, and 1.28 times that of the plain configured FPSAH system with reflector. Figure 10 displays the overall outcomes of thermal performance in terms of *Nu* for different configurations.

A higher rise in heat release rate was observed with the artificially roughened FPSAH system using radiation reflectors. This may be due to the random flow behavior of air inside the duct and increased heat exposure to the absorber surface. The use of radiation reflectors not only increases the temperature of the absorber surface but also enhances heat loss to the atmosphere. Therefore, it is necessary to transfer maximum heat from the surface to the flowing fluid.

Artificial roughness diminishes thermal boundary layer formation near the absorber surface by creating more turbulence inside the duct. W-shaped rib roughness develops a critical flow pattern attributed to the formation of secondary flows along the ribs and flow reattachment in the inter-rib region. The combination of artificial roughness and radiation reflectors results in substantial enhancement in the FPSAH system.

**Fig. 11:**
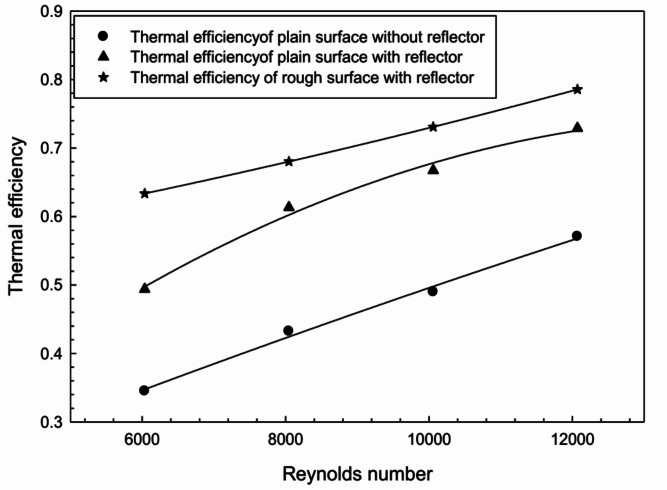
Thermal efficiency of different sets used in FPSAH system.

To evaluate the thermal characteristics of different FPSAH configurations, Fig. 11 shows the thermal productivity of the system corresponding to various Reynolds numbers. Higher thermal efficiency is achieved by the system with a roughened absorber surface and radiation reflector. The thermal efficiency ranges from 1.37 to 1.84 times that of the system without reflector and roughness.

### Machine learning application

**Table 3:** Experimental sets used in machine learning.

Case	V (m/s)	$T_i$ (°C)	$T_o$ (°C)	$T_p$ (°C)	Re	$Nu_s$	*Nu*	$f_s$	*f*
Smooth surface only	2.1	29.52	32.69	60.09	6036	21.22	20.71	0.0096	0.0099
	2.8	29.84	32.82	59.21	8045	26.72	26.98	0.0089	0.0091
	3.5	29.88	32.61	57.31	10060	31.94	33.05	0.0084	0.0086
	4.2	30.06	32.71	56.54	12072	36.95	39.89	0.0080	0.0082
Smooth surface with reflector	2.1	29.44	33.86	64.97	6036	21.22	25.11	0.0096	0.0308
	2.8	29.55	33.71	62.67	8045	26.72	33.83	0.0089	0.0267
	3.5	29.93	33.61	61.01	10060	31.94	39.71	0.0084	0.0261
	4.2	30.17	33.52	58.56	12072	36.95	47.48	0.0080	0.0258
Roughened surface with reflector	2.1	28.85	34.55	65.35	6036	21.22	32.07	0.0096	0.0308
	2.8	29.04	33.68	62.12	8045	26.72	38.08	0.0089	0.0267
	3.5	29.13	33.16	57.46	10060	31.94	48.32	0.0084	0.0261
	4.2	29.25	32.86	53.54	12072	36.95	60.79	0.0080	0.0258

In this part, two machine learning (ML) algorithms were used to predict *Nu* values for three experimental configurations: smooth surface only, smooth surface with reflector, and roughened surface with reflector, as shown in Table 3. Random Forest and Robust Regression were applied using Python programming on the Google Colaboratory platform.

The performance of the ML models was evaluated using metrics such as Mean Squared Error (MSE), Mean Absolute Error (MAE), Root Mean Squared Error (RMSE), and R-squared ($R^2$) value. Table 4 shows the results for both regression methods. Random Forest Regression achieved a curve fitting of 0.94 for the testing dataset, while Robust Regression provided a better fit with a curve fitting of 0.99.

**Table 4:** Estimation of testing and training using machine learning algorithms.

Algorithm	Testing				Training			
	MAE	MSE	RMSE	$R^2$	MAE	MSE	RMSE	$R^2$
Robust regression	1.94	9.18	3.02	0.93	0.01	5.60	0.01	0.99
Random forest regression	3.65	20.63	4.54	0.69	1.65	5.33	2.31	0.94

### Artificial neural network (ANN)

A total of 610 experimental results were used to train the ANN model, split into three sets: 412 training data, 104 test data, and 94 validation data. Selecting the appropriate ANN architecture is crucial due to its significant influence on prediction accuracy^46,47^.

**Fig. 12:**
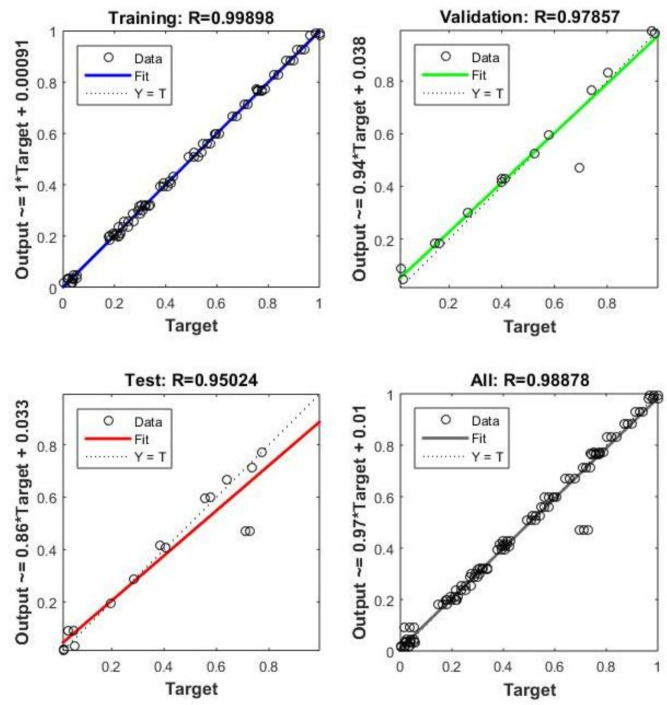
Hidden layer’s optimal performance was determined to be 30 neurons.

After evaluating various neural network configurations, the model was optimized by minimizing MSE. Figure 12 shows the 30-neuron ANN structure used for *Nu* prediction. The Levenberg-Marquardt learning algorithm and a linear transfer function were used in the output layer.

**Fig. 13:**
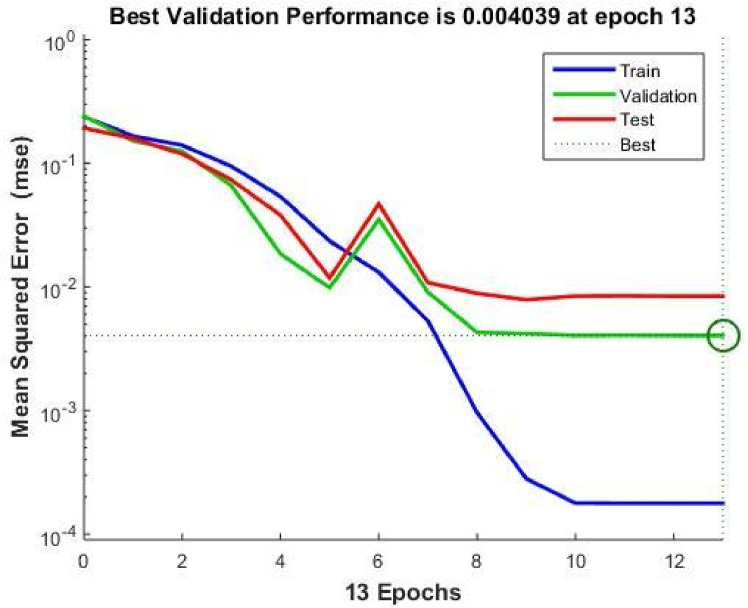
Number of iterations.

Tables 5 and 6 show the correlation coefficients for training, testing, and validation datasets. Figure 13 illustrates the total number of iterations completed during training.

**Table 5:** ANN performance for Nusselt number prediction.

HL	Neurons	Train $R^2$	Val $R^2$	Test $R^2$	All $R^2$
1	10	0.93641	0.99990	0.99991	0.94921
1	15	0.93643	0.99992	0.99993	0.94923
1	20	0.93647	0.99994	0.99995	0.94935
1	25	0.93655	0.99996	0.99997	0.94939
1	30	0.93661	1.00000	1.00000	0.94941
1	40	0.93658	0.99994	0.99998	0.94938

**Table 6:** ANN performance for thermal efficiency prediction.

HL	Neurons	Train $R^2$	Val $R^2$	Test $R^2$	All $R^2$
1	10	0.83202	0.99991	0.99992	0.87158
1	15	0.83205	0.99995	0.99994	0.87160
1	20	0.83206	0.99996	0.99996	0.87161
1	30	0.83209	0.99997	0.99997	0.87163
1	40	0.83212	1.00000	1.00000	0.87165
1	50	0.83208	0.99996	0.99996	0.87164

**Fig. 14:**
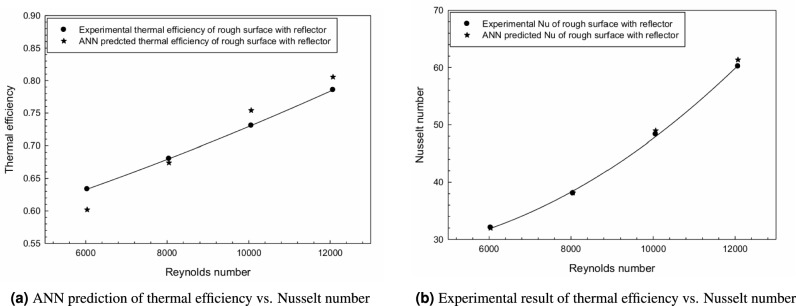
Comparative analysis of thermal efficiency and Nusselt number: (**a**) ANN prediction results; (**b**) Experimental validation results.

Figure  14a and b show the comparison between thermal efficiency and Nusselt number of rough surfaces with reflectors, evaluated using the ANN model and experimental results respectively.

## Error analysis

Different error metrics have been used to evaluate the results^48^. The detailed formulae are provided below.

### Mean absolute error

The sum of all absolute discrepancies between the expected and observed values. It can be evaluated using

16 $$\begin{aligned} \text {MAE} = \frac{1}{n}\sum _{i=1}^{n}|y_i - x_i| \end{aligned}$$

### Root mean square error

It is a square root of MSE, which brings metric back to the same unit as the target variable, and it can be mathematical written as

17 $$\begin{aligned} \text {RMSE} = \sqrt{\frac{1}{n}\sum _{i=1}^{n}(y_i - x_i)^2} \end{aligned}$$

### Mean square error

The sum of the squared discrepancies between the expected and observed values^49^. It can be calculated using

18 $$\begin{aligned} \text {MSE} = \frac{1}{n}\sum _{i=1}^{n}(y_i - x_i)^2 \end{aligned}$$

### R-square

It shows how dependent variable’s volatility can be predicted by independent variables.

19 $$\begin{aligned} R^2 = 1 - \frac{S_{\text {res}}}{S_{\text {tot}}} \end{aligned}$$

where $y_i$ is predicted value and $x_i$ is an actual value. $S_{\text {res}}$ is sum of residue and $S_{\text {tot}}$ is sum of total squares.

### Correlation

#### Functional relationship of correlations

Experimental analysis and effects of various roughness parameters and Reynolds number (Re) on thermal features is discussed to develop the statistical correlations that cover all the geometric and flow factors. Various rough parameters have a strong effect on Nusselt number and can be declared in form of functional relationship shown in Eq. (20).

20 $$\begin{aligned} \text {Nu} = f\left( \text {Re}, \frac{h}{D_h}, \frac{q}{h}\right) \end{aligned}$$

#### Correlation for Nusselt number

To determine functional connections a regression analysis is performed. Nu has created statistical correlations that match the experimental outcomes of every plate that was examined.

**Fig. 15:**
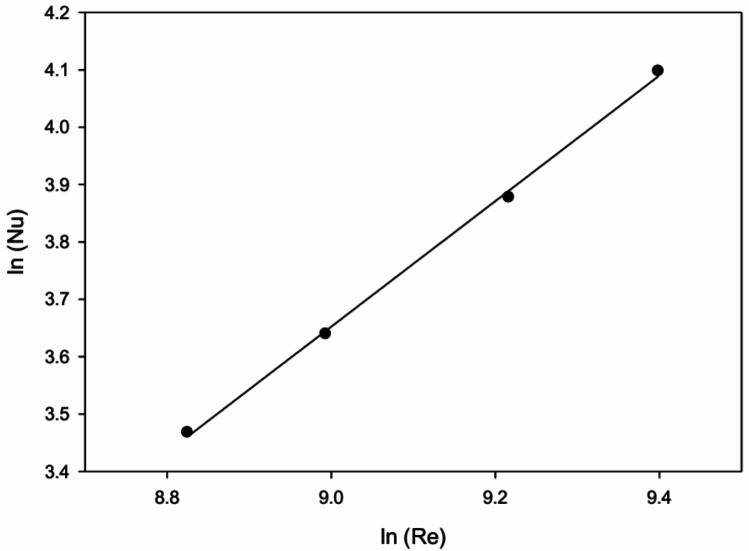
Plot of ln (Nu) in terms of ln (Re).

Obtained value of Nu and Re are plotted on log-log scale for recorded data of all plates and is shown in Fig. 15. Analysis of regression to linearly fit the curve going through the points specified by an Eq. (21).

21 $$\begin{aligned} \ln (\text {Nu}) = C_1 + n \ln (\text {Re}) \end{aligned}$$

22 $$\begin{aligned} \text {Nu} = A_o (\text {Re})^n \end{aligned}$$

The acquired value of $A_o$ is now dependent on additional geometrical factors related to roughness. Hence, $h/D_h$ & *q*/*h* is considered as the next parameter and plotted for the regression analysis. The points are fitted by using second order polynomial passing through the points obtained to get correlation for ‘Nu’.

23 $$\begin{aligned} \begin{aligned} \text {Nu} = 0.004(\text {Re})^{1.0944}\left( \frac{h}{D_h}\right) ^{0.02}\left( \frac{q}{h}\right) ^{0.0301} \\ \times \exp \left[ -0.12\left( \ln \left( \frac{q}{h}\right) \right) ^2\right] \end{aligned} \end{aligned}$$

**Fig. 16:**
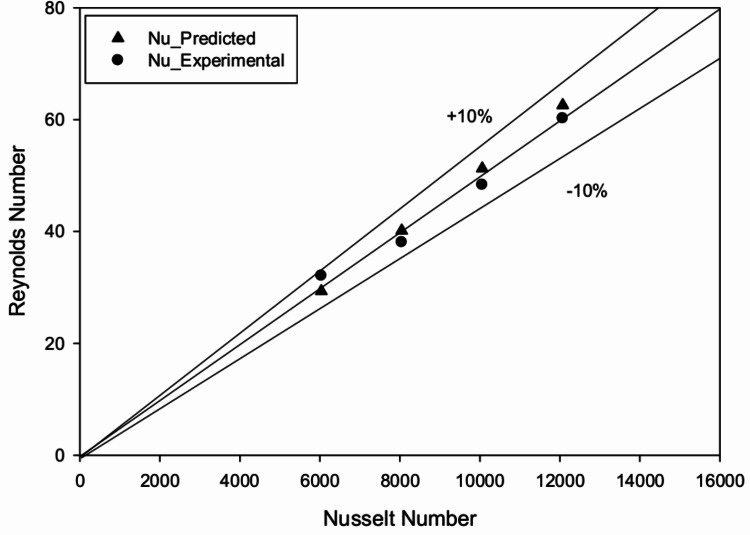
Comparison of experimental Nusselt number against predicted Nusselt number from correlation.

Figure 16 shows a comparison between experimental ‘Nu’ and that predicted from correlation equations. It is realized that almost all data points lie within the deviance line ± 10%. Hence correlation developed is sensibly acceptable for predicting Nu of roughened solar heater with the parameter range investigated.

## Conclusions

Based on the experimental study made on rectangular passage of FPSAH system with roughened broad wall at one side, thermal characteristics were investigated. The rectangular duct of the FPSAH system is tested under different combinations of applying radiation reflectors at the edges along with roughened absorber surface. Additionally, artificial neural network (ANN) and machine learning (ML) algorithms were used to forecast Reynolds number in each set of experiments. The following conclusions have been drawn from the present study.

1. Incorporation of radiation reflectors at edges of the FPSAH system expressively augmented temperature of absorber surface as compared to system without radiation reflectors by redirecting and concentrating additional incident solar radiation onto the absorber surface, thereby reducing optical losses and improving solar energy utilization.
2. An increase in ‘Nu’ has been observed with simultaneous use of W-shaped artificial roughness along with radiation reflectors as W shaped roughness element effectively promoted the disorder and improves convective heat discharge. The combined effect of improved disorder and amplified concentration of solar radiations leads to a significant improvement in thermal performance of FPSAH system.
3. The FPSAH system configured with W-shaped artificial roughness and radiation reflectors gives remarkable augmentation in Nusselt number as 1.62 times over plain configuration of absorber plate.
4. The dual mode of FPSAH system exhibit a significant improvement in thermal efficiency, recorded an augmentation of approximately 1.84 times over the plain configuration of the system.
5. Machine Learning (ML) algorithms can be effectively applied to predict the Reynolds number based on relevant operating and geometrical paremeters. Amongst different ML module, Robust Regression is found to be the best suitable algorithm for estimating the Re value under different experimental conditions.
6. The use of Artificial Neural Network (ANN) reveals high accurate prediction capability for the experimental data with an acceptable coefficient value of 1.00, that confirms the effectiveness of applying ANN model in the fundamental association amongst input parameters and system performance.

In addition, optimal ANN model and ML algorithm results are found to be more accurate than predicted results from published correlations. To summarize, this technique has the potential to contribute to a better understanding of heat exchanger dynamic behavior in the future.

The solar air heater can be used for space heating in homes, greenhouses, and agricultural settings. The warm air generated can be circulated to maintain comfortable indoor temperatures or promote plant growth. Additionally, can be also implemented in industries that require moderate heating, such as drying processes in food, textiles, or lumber, and can benefit from the efficient heat generated by solar air heaters.

## Data Availability

The datasets generated and/or analyzed during the current study are available from the corresponding author upon reasonable request.

## List of symbols

$A_p$: Area of heated plate (m$^2$)
$A_o$: Cross-sectional area of orifice plate (m$^2$)
$C_p$: Specific heat of fluid (kJ kg$^{-1}$ K$^{-1}$)
$C_d$: Orifice discharge coefficient
$D_h$: Hydraulic diameter of rectangular duct (m)
*p*: Roughness height (mm)
*H*: Rectangular duct height (mm)
$h_t$: Convective heat transfer coefficient (W m$^{-2}$ K$^{-1}$)
*k*: Thermal conductivity of flowing air (W m$^{-1}$ K$^{-1}$)
*L*: Absorber plate length (mm)
$\dot{m}$: Mass flow rate of air (kg s$^{-1}$)
*q*: Roughness pitch (mm)
$Q_u$: Useful heat gain (W)
$T_f$: Mean air temperature (K)
$T_i$: Inlet air temperature (K)
$T_o$: Outlet air temperature (K)
$T_p$: Average heated plate temperature (K)
*V*: Air velocity (m s$$^{-1}$$)
*W*: Duct width (mm)

## Dimensionless parameters

$h/D_h$: Relative roughness height
Nu: Nusselt number (rough plate)
Nu$_s$: Nusselt number (smooth plate)
*q*/*h*: Relative roughness pitch
Pr: Prandtl number
Re: Reynolds number
*W*/*H*: Aspect ratio

## Greek symbols

$\alpha$: Angle of attack (degree)
$\rho _{\text {air}}$: Density of air (kg m$^{-3}$)
$$\nu$$: Kinematic viscosity (m$$^2$$ s$$^{-1}$$)
$$\eta _{th}$$: Thermal efficiency
